# Effect of Fe on Co-Based SiO_2_Al_2_O_3_ Mixed Support Catalyst for Fischer–Tropsch Synthesis in 3D-Printed SS Microchannel Microreactor

**DOI:** 10.3390/molecules30173486

**Published:** 2025-08-25

**Authors:** Meric Arslan, Sujoy Bepari, Juvairia Shajahan, Saif Hassan, Debasish Kuila

**Affiliations:** 1 Department of Applied Science and Technology, North Carolina Agricultural and Technical State University, Greensboro, NC 27411, USA; marslan@ncat.edu; 2 Department of Chemistry, North Carolina Agricultural and Technical State University, Greensboro, NC 27411, USA; 3 Joint School of Nanoscience and Nanoengineering, North Carolina Agricultural and Technical State University, Greensboro, NC 27411, USA

**Keywords:** Fischer–Tropsch synthesis, stainless steel microreactor, co catalyst, Fe catalyst, silica–alumina support, mesoporous composite oxide

## Abstract

This research explores the effect of a composite support of SiO_2_ and Al_2_O_3_ with Fe and Co incorporated as catalysts for Fischer–Tropsch synthesis (FTS) using a 3D-printed stainless steel (SS) microchannel microreactor. Two mesoporous catalysts, FeCo/SiO_2_Al_2_O_3_ and Co/SiO_2_Al_2_O_3_, were synthesized via a one-pot (OP) method and extensively characterized using N_2_ physisorption, XRD, SEM, TEM, H_2_-TPR, TGA-DSC, FTIR, and XPS. H_2_-TPR results revealed that the synthesis method significantly affected the reducibility of metal oxides, thereby influencing the formation of active FTS sites. SEM-EDS and TEM further revealed a well-defined hexagonal matrix with a porous surface morphology and uniform metal ion distribution. FTS reactions, carried out in the 200–350 °C temperature range at 20 bar with a H_2_/CO molar ratio of 2:1, exhibited the highest activity for FeCo/SiO_2_Al_2_O_3_, with up to 80% CO conversion. Long-term stability was evaluated by monitoring the catalyst performance for 30 h on stream at 320 °C under identical reaction conditions. The catalyst was initially active for the methanation reaction for up to 15 h, after which the selectivity for CH_4_ declined. Correspondingly, the C_4_^+^ selectivity increased after 15 h of time-on-stream, indicating a shift in the product distribution toward longer-chain hydrocarbons. This trend suggests that the catalyst undergoes gradual activation or restructuring under reaction conditions, which enhances chain growth over time. The increase in C_4_^+^ products may be attributed to the stabilization of the active sites and suppression of methane or light hydrocarbon formation.

## 1. Introduction

Fischer–Tropsch synthesis (FTS) involves conversion of synthesis gas (syngas), a mixture of hydrogen and carbon monoxide, into long-chain hydrocarbons. The process is named after Franz Fischer and Hans Tropsch, who invented hydrocarbon production using cobalt catalysts in 1923 [1]. Syngas can be derived from various carbon-based feedstocks, including natural gas, biogas, biomass, coal, and waste, using different reforming techniques [2,3,4,5,6,7]. This can involve both single- and two-step processes [8,9,10]. For example, syngas can be produced in the presence of oxygen and water through coal gasification [11]. The selectivity of products in FTS plays a pivotal role in its viability, as the formation of byproducts such as CH_4_ and CO_2_ is not desirable. Therefore, there is a tremendous interest in the development of stable catalysts for FTS to improve product selectivity for the economical production of hydrocarbons [12].

In Fischer–Tropsch synthesis (FTS), the commonly used active metals are cobalt (Co), iron (Fe), and ruthenium (Ru), due to their high catalytic activity and selectivity under different reaction conditions [13,14]. Iron (Fe)-based catalysts offer several distinct advantages over other metals for large-scale industrial applications: (i) low cost and abundant availability, making them economically favorable; (ii) intrinsic promotion of the water gas shift (WGS) reaction, which is particularly beneficial for syngas derived from coal or biomass with low H_2_/CO ratios; and (iii) high thermal stability and activation temperature, with a product distribution that favors short-chain olefins, oxygenates, and gasoline-range hydrocarbons [15,16,17,18]. Support materials play an important role in enhancing the performance of Fe-based catalysts, and mesoporous support with large surface areas are often incorporated in FTS. These supports not only improve the mechanical strength and dispersion of the active phase, but also the overall catalytic activity by increasing the surface area and providing more accessible active sites [19,20,21]. The support materials for FTS usually include SiO_2_, ZnO, TiO_2_, ZrO_2_, and γ-Al_2_O_3_ [22,23,24,25]. A significant drawback of many of these oxide supports is the formation of mixed metal support oxides during catalyst preparation due to the strong interaction between the metal and the supports. These mixed oxides are often difficult to reduce, which limits the availability of the active metallic phase and thereby reduces the overall catalytic activity in the FTS process.

In addition, obtaining a high loading of active metals on such supports is challenging, further limiting the catalyst performance in FTS processes [19]. For example, silica-supported Fe-based catalysts tend to form iron silicate, which cannot be fully reduced to active α-Fe, as reported by Wielers et al. [26]. Similarly, Zhang et al. [27] observed that iron silicate formation hinders the iron oxide reduction, resulting in decreased FTS activity. Therefore, modifying the Fe/SiO_2_ interaction is desirable to enhance both the stability and catalytic performance [28,29,30,31]. An alternative option to overcome the stability and activity issues of the catalysts is to consider catalysts in which the support can protect the metal from sintering or deactivation. Ni et al. [32,33,34] studied Fe-based catalysis with promoters such as potassium and graphite carbon (GC) for the FTS reaction. They reported that GC and potassium additions significantly enhanced catalytic activity. While GC acted as a reductant, facilitating the reduction of Fe^3+^ to metallic Fe^0^ while also strengthening the SiO_2_ channels, potassium increased CO chemisorption but inhibited H_2_ chemisorption, which lowered hydrogenation capacity and boosted olefin selectivity. In another study, Ni et al. [28] modified an Fe@SiO_2_ catalyst with GC for FTO. The incorporation of GC altered the Fe-SiO_2_ interaction, and the rigid porous framework of GC helped maintain open channels for syngas access, preventing mesopore collapse during the reaction.

In our previous study, we used a Co-based SiO_2_Al_2_O_3_ catalyst for FTS [35]. The catalyst achieved 45% CO conversion. In this study, we focused on the influence of their physical and chemical properties; specifically, Fe addition with Co on SiO_2_ Al_2_O_3_ mixed oxide for Fischer–Tropsch synthesis (FTS) [36]. Iron-based catalysts were synthesized using the one-pot (OP) method, and their catalytic performances were evaluated in 3D-printed stainless steel microreactors at 20 bar. We investigated the effect of the SiO_2_-Al_2_O_3_ support by incorporating Al_2_O_3_ on SiO_2_ and the bimetallic FeCO core. The use of a SiO_2_-Al_2_O_3_ mixed oxide support offered the synergistic effect of properties from both pure oxide supports. SiO_2_ provided high surface area and thermal stability, while Al_2_O_3_ contributed to enhanced metal–support interactions and moderate acidity, which could influence the distribution and reducibility of the active metal phase. Compared to pure oxides, the mixed support could improve catalyst performance by optimizing the balance between metal dispersion, reducibility, and support acidity, ultimately enhancing catalytic activity and selectivity in FTS. In our previous work, we studied a SiO_2_-Al_2_O_3_ support for the FTS process [37]. The catalysts were characterized by N_2_ adsorption–desorption, XRD, SEM-EDS, TEM, H_2_-TPR, FTIR, TGA-DSC, and XPS before the FTS reactions to understand the effects of adding Fe metal with Co on a SiO_2_Al_2_O_3_ support on the product selectivity and CO conversion of the reaction.

## 2. Experimental Section

### 2.1. Materials

All chemicals used in this study were of analytical grade and employed without any further purification. Tetraethyl orthosilicate (TEOS, 99%) and ammonium hydroxide were sourced from Acros Organics (Fair Lawn, NJ, USA). Iron (III) nitrate nonahydrate (Fe (NO_3_)_3_·9H_2_O), cobalt (II) nitrate hexahydrate (Co (NO_3_)_2_·6H_2_O), and cetyltrimethylammonium bromide (CTAB) were obtained from Sigma-Aldrich (Burlington, MA, USA). Ethanol, absolute ethanol, and acetone were purchased from Fisher Scientific (Fair Lawn, NJ, USA).

### 2.2. Synthesis of FeCo/SiO_2_Al_2_O_3_ Catalyst

Bimetallic FeCo catalysts supported on SiO_2_–Al_2_O_3_ were synthesized via a one-pot (OP) sol–gel method adapted from previously reported procedures, Supporting Information). In a typical synthesis, CTAB was dissolved in deionized (DI) water at 35 °C and stirred until fully dissolved. The required quantities of iron and cobalt nitrates, calculated based on the target metal loading, were dissolved in absolute ethanol to form a clear metal precursor solution. The solution was stirred for 30 min to ensure homogeneity.

The metal nitrate solution was then added dropwise to the CTAB solution under continuous stirring to enable the formation of metal–surfactant micellar complexes. After an additional 30 min of stirring, TEOS was added dropwise as the silica source while maintaining a stirring speed of 350 rpm. The mixture was stirred for 30 min to facilitate partial hydrolysis and condensation of TEOS.

To initiate co-precipitation and further drive the sol–gel reaction, ammonium hydroxide was added dropwise to the reaction mixture while maintaining the pH between 9 and 10. The resulting suspension was stirred at ambient temperature for 20 h to allow for complete hydrolysis of TEOS and incorporation of the metal hydroxides into the silica–alumina matrix.

The precipitate was collected by filtration and washed repeatedly with DI water until the filtrate reached neutral pH (~7) to remove excess surfactant and residual precursors. This was followed by washing with ethanol to remove organics and to accelerate drying. The solid was air-dried at room temperature for 24 h and further dried in an oven at 98 °C for an additional 24 h.

Calcination was performed in air at 550 °C for 6 h at a heating rate of 2 °C min^−1^ to decompose the surfactant and stabilize the mesoporous structure. The calcined powders were allowed to cool to room temperature in a furnace before further characterization and catalytic testing.

### 2.3. The 3D-Printed SS Microchannel Microreactor

For high-pressure Fischer–Tropsch synthesis, a microchannel reactor was used with secured sealing at the inlet and outlet, under high-pressure conditions. The AutoCAD 23.0 schematic and the final stainless steel 3D-printed reactor are presented in reference [38]. The cross-sectional view illustrates seven parallel microchannels, each 5 cm long and 1000 µm wide and deep, connecting the cylindrical inlet and outlet sections. Both the inlet and outlet were engineered with outer diameters compatible with ¼-inch Swagelok tubing, allowing for precise coupling with a Swagelok filter to achieve robust, leak-proof sealing during high-pressure operation [38].

### 2.4. Characterization

Nitrogen adsorption–desorption isotherms were measured at −196 °C using a BET surface area analyzer (3Flex, Micromeritics, Norcross, GA, USA). Specific surface areas were calculated by the Brunauer–Emmett–Teller (BET) method, while pore size distributions were determined using the Barrett–Joyner–Halenda (BJH) method.

X-ray diffraction (XRD) patterns were collected using a Bruker AXS powder (Boston, MA, USA) diffractometer with Cu Kα_1_ radiation (λ = 1.5406 Å) over a 2θ range of 10° to 70°, employing a step size of 0.02°. Phase identification and crystalline grain size were determined from the diffraction profiles.

Temperature-programmed reduction (TPR) experiments were conducted on a 3Flex chemisorption analyzer (Micromeritics, Norcross, GA, USA). Approximately 50 mg of catalyst was loaded into a quartz tube reactor, secured between a quartz wool plug and a quartz frit. The sample was exposed to 10% H_2_ in Ar (*v*/*v* = 1:9) at a flow rate of 50 mL min^−1^, and heated from room temperature to 800 °C at a ramp rate of 10 °C min^−1^ to assess metal oxide reducibility.

Morphological features and surface topography were analyzed using a ZEISS (White Plains, NY, USA) Auriga Focused Ion Beam Scanning Electron Microscope (FIB-SEM) at the Joint School of Nanoscience and Nanoengineering. Particle distribution and crystallinity were evaluated by transmission electron microscopy (TEM) using a Thermo-Fisher Talos F200X instrument (Hillsboro, Oregon, USA) operated at 200 kV.

Surface chemical composition and oxidation states were determined via X-ray photoelectron spectroscopy (XPS) using a Thermo Scientific Escalab Xi+ spectrometer (West Sussex, UK). Surface functional groups were identified by Fourier transform infrared spectroscopy (FTIR) using a Shimadzu IR Prestige-21 instrument (Columbia, MD, USA) equipped with a mercury–cadmium–telluride (MCT) detector.

Thermogravimetric analysis coupled with differential scanning calorimetry (TGA-DSC) was performed on a TA Instruments system (New Castle, DE, USA) to investigate the thermal stability and decomposition behavior of the polymer components of the catalyst precursor.

### 2.5. FTS Process

**Figure 1:**
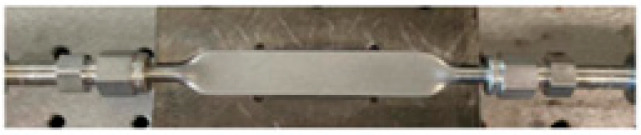
The 3D-printed stainless steel microchannel microreactor [38].

The catalyst was introduced into the microreactor using a packing procedure designed to optimize the utilization of the available microchannel volume. The quantity of catalysts used for FTS was determined by weighing the microreactor before and after loading, resulting in a net catalyst mass of 0.16 g. To prevent catalyst loss during operation, both the inlet and outlet of the microreactor were sealed using ¼-inch Swagelok VCR filter gaskets, to ensure secure containment and compatibility with high-pressure conditions. The catalyst-loaded microreactor was then mounted into a custom-fabricated reactor block (see Figure 1) and integrated into the reaction system using ¼-inch female Swagelok fittings for leak-tight and pressure-stable operation [38,39].

FTS experiments were performed using a custom-designed, LabVIEW-automated (Austin, TX, USA) system that enabled precise control over flow rates, temperature, and pressure, as shown elsewhere [35]. The syngas mixture, consisting of H_2_ and CO in a 2:1 molar ratio, was introduced into the reactor using a Bronkhorst mass flow controller with a maximum capacity of 20 sccm. Nitrogen, used as a carrier and internal standard, was supplied via a Bronkhorst mass flow controller (Bethlehem, PA, USA) with 20 sccm capacity.

The upstream and downstream pressures were continuously monitored using Bronkhorst pressure sensors (Bethlehem, PA, USA). The readings were relayed to a Bronkhorst solenoid valve for real-time pressure regulation. The entire system was operated using a custom LabVIEW 2018 interface for automated control and data acquisition.

Prior to catalytic testing, the metal oxide-based catalyst was subjected to in situ reduction under hydrogen flow at 350 °C overnight inside the microreactor to activate the metal sites of the catalysts at 1 bar. After the reduction process, the microreactor temperature was reduced to the desired reaction temperature in the presence of N_2_ flow to maintain an inert atmosphere for the reduced catalyst. The microreactor temperature was measured using a thermocouple. The FTS reactions were carried out at a constant gas hourly space velocity (GHSV) of 12,000 h^−1^ in the temperature range of 200 to 380 °C at 20 bar. The syngas flow rates were maintained at 4 mL/min for H_2_ and 2 mL/min for CO, while the N_2_ flow was held constant at 1.5 mL/min. To obtain reproducible data, the FTS reaction was carried out three times at each temperature.

The reaction products were analyzed both qualitatively and quantitatively using a combination of gas chromatography (GC) and mass spectrometry (MS). Specifically, an Agilent 7890B gas chromatograph coupled with a 5977 MSD detector (Santa Clara, CA, USA) was employed for product identification and quantification [35].

## 3. Result and Discussion

### 3.1. Brunauer–Emmett–Teller (BET) Analysis

**Figure 2:**
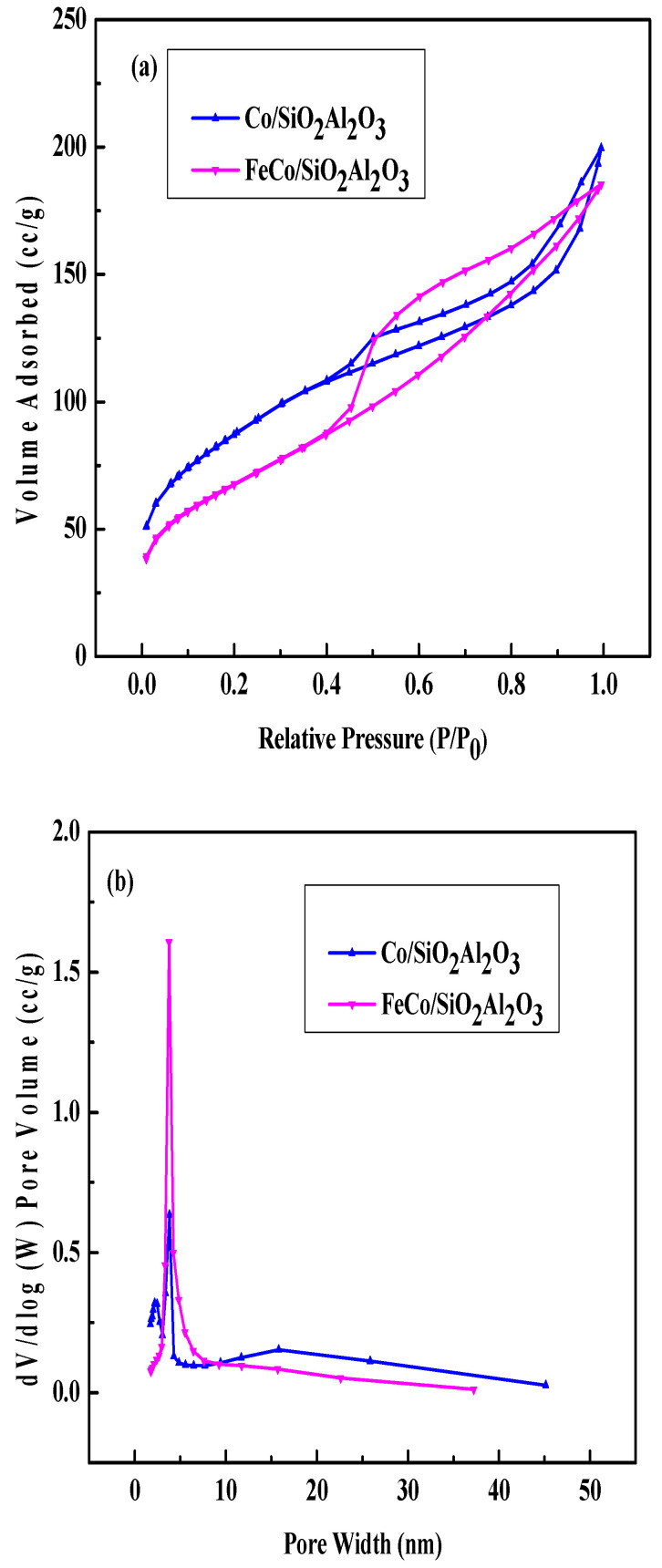
N_2_ adsorption–desorption: (**a**) isotherms and (**b**) pore size distribution plots of Co/SiO_2_Al_2_O_3_ and FeCo/SiO_2_Al_2_O_3_ catalysts.

To evaluate the textural properties of the synthesized catalysts, nitrogen adsorption–desorption measurements were performed using the BET method. Capillary condensation was observed in the relative pressure (P/P_0_) range of 0.6–1.0. The isotherms of Co/SiO_2_Al_2_O_3_ and FeCo/SiO_2_Al_2_O_3_ exhibited Type IIb behavior with an H3 hysteresis loop, indicative of interparticle pores formed by aggregated particles (Figure 2a) [35,40]. Capillary condensation in the P/P_0_ range of 0.45–1.0 further supports the presence of mesoporous structures in these catalysts. The BJH pore size distribution plots derived from the desorption branch (Figure 2b) show a sharp peak at 3.76 nm for both catalysts, confirming the presence of mesopores.

A notable reduction in surface area was observed for the Co/SiO_2_Al_2_O_3_ and FeCo/SiO_2_Al_2_O_3_ catalysts, which is attributed to the incorporation of Al_2_O_3_ into the mesoporous SiO_2_ framework within the catalyst architecture [39].

Table 1 shows the surface area, pore volume, and pore diameter of the two catalysts. The surface area decreased with the addition of Fe. The decrease in surface area was supported by the increase in pore volume and pore diameter.

### 3.2. X-Ray Diffraction (XRD) Analysis

**Figure 3:**
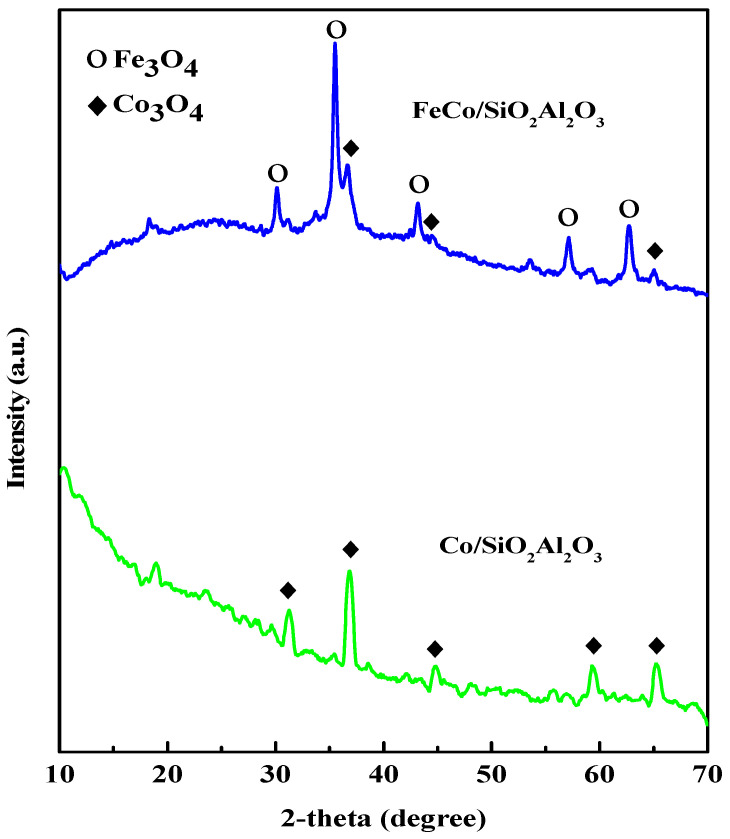
Wide-angle XRD patterns of FeCo/SiO_2_Al_2_O_3_ and Co/SiO_2_Al_2_O_3_ catalysts.

The X-ray diffraction (XRD) patterns of the samples are shown in Figure 3.

XRD analysis showed the presence of a cubic Co_3_O_4_ phase in the Co/SiO_2_Al_2_O_3_ catalyst, with characteristic diffraction peaks observed at 2θ values of 31.27° (220), 36.54° (311), 44.57° (400), 59.41° (511), and 64.90° (440). These results are consistent with the standard JCPDS-42-1467 pattern (Zinc Oxide (ZnO). International Centre for Diffraction Data: Newtown Square, PA, USA, 1991) of Co_3_O_4_ reported in the literature [41]. For the FeCo/SiO_2_Al_2_O_3_ catalyst, the diffraction peaks indicate the presence of both Co_3_O_4_ and Fe_3_O_4_ crystalline phases, exhibiting cubic and orthorhombic structures, respectively. The Fe_3_O_4_ phase was identified by peaks at 2θ values of 30.20° (200), 35.46° (121), 43.09° (004), 57.09° (321), and 62.71° (400), corresponding to JCPDS-75-1609 [41]. The Co_3_O_4_ peaks at 36.54° (311), 44.57° (400), and 64.90° (440) were also observed for the FeCo/SiO_2_Al_2_O_3_ catalyst, confirming its coexistence. Notably, the characteristic SiO_2_ peak was absent in the XRD patterns of both Co/SiO_2_Al_2_O_3_ and FeCo/SiO_2_Al_2_O_3_ catalysts, likely due to the incorporation of Al_2_O_3_ into the mesoporous silica shell, which may have altered the structural ordering [42]. The average crystallite size of each catalyst was calculated from the XRD data using the modified Scherrer equation [43], and the results are presented in Table 2. Comparable crystallite sizes were observed for both catalysts. According to our previous study, the average crystallite size of Co_3_O_4_ in the Co/SiO_2_Al_2_O_3_ catalyst was 10.36 nm [35]. In contrast, the Co_3_O_4_ crystallite size in the FeCo/SiO_2_Al_2_O_3_ catalyst increased to 16.35 nm. This increase in size may be attributed to the incorporation of iron oxide or its interaction with cobalt oxide during the synthesis process, which could influence particle aggregation or crystallization behavior in the SEM images described below.

### 3.3. Scanning Electron Microscopy (SEM) Analysis

**Figure 4:**
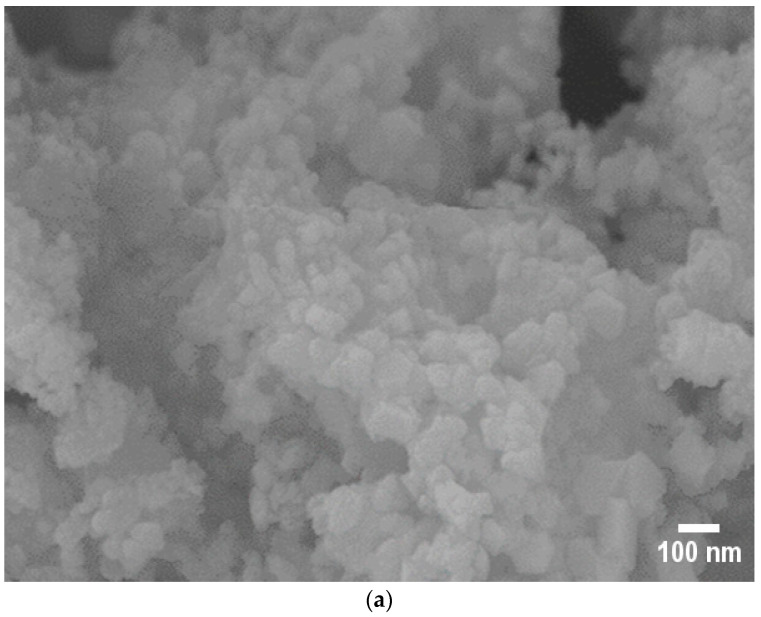

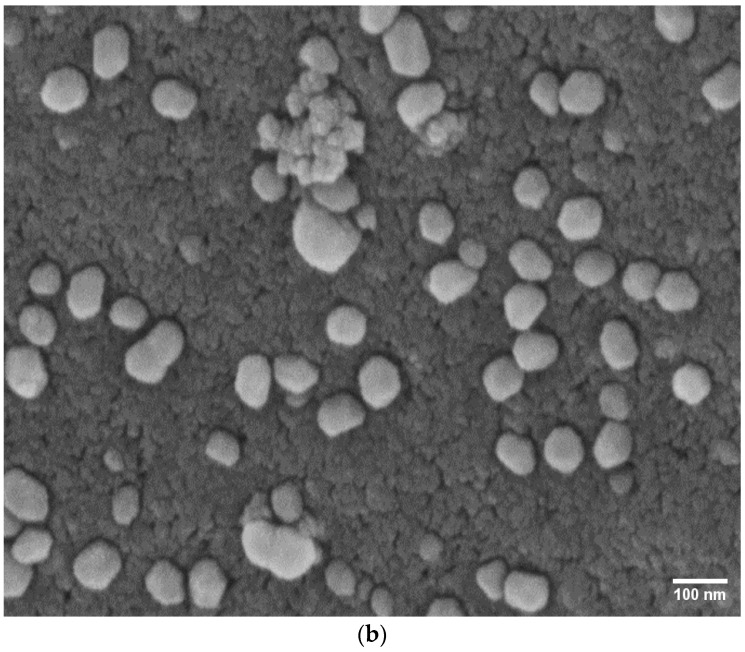
Scanning Electron Microscopy (SEM) images of (**a**) Co/SiO_2_Al_2_O_3_ and (**b**) FeCo/SiO_2_Al_2_O_3_ catalysts.

The morphologies and elemental compositions (wt.%) of the catalysts were characterized using Scanning Electron Microscopy (SEM). The SEM images of the catalysts are presented in Figure 4. Noticeable agglomeration of particles was observed in the Co/SiO_2_Al_2_O_3_ (OP) catalyst (Figure 4a) [35]. This may be attributed to the presence of Al_2_O_3_ in the mesoporous silica. This incorporation likely reduced the adhesion of cobalt to the support, promoting aggregation [44]. In contrast, the presence of iron (Fe) with Co significantly altered the morphology of the FeCo/SiO_2_Al_2_O_3_ catalyst (Figure 4b), resulting in more uniform dispersion of particles throughout the support matrix.

Table 3 presents the energy-dispersive X-ray spectroscopy (EDS) results for all the catalysts [45]. In our previous work, the addition of Al_2_O_3_ with the SiO_2_ support Co/SiO_2_Al_2_O_3_ (OP) catalyst altered the metal loading. Similarly, in the current study, the incorporation of Fe into the FeCo/SiO_2_Al_2_O_3_ catalyst influenced the distribution of other metals, resulting in an increased Co content and decreased loadings of Si and Al. The detected oxygen (O) was attributed to surface oxidation of the metals in all catalysts. EDS analysis was performed on five randomly selected regions for each catalyst sample [35].

### 3.4. H_2_ Temperature Programmed Reduction (TPR) Analysis

**Figure 5:**
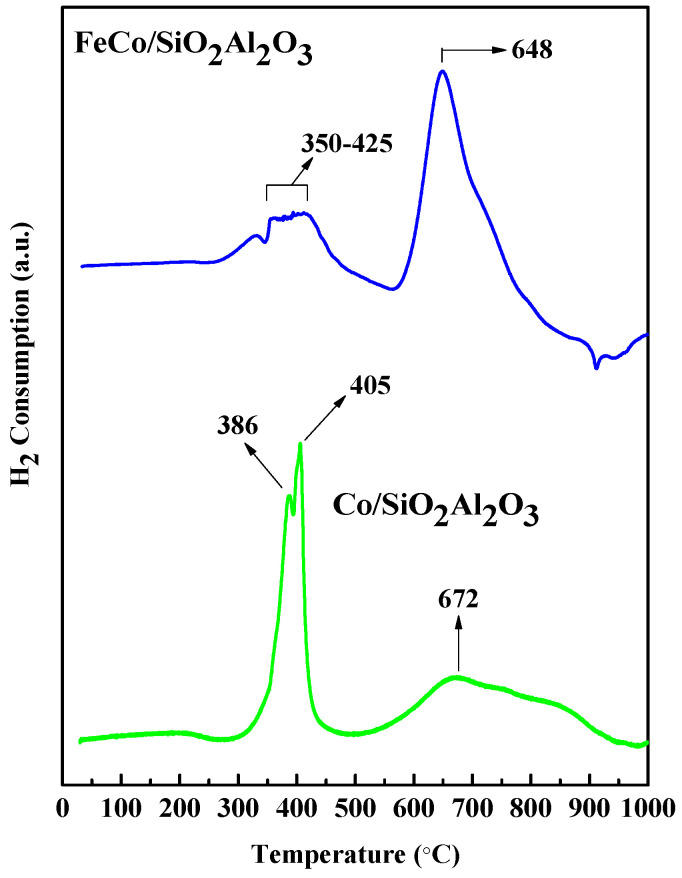
H_2_−TPR analyses of all catalysts.

Temperature programmed reduction (TPR) with H_2_ was conducted to examine the reduction behavior of metal oxides and metal–support interactions that may have played a significant role. The H_2_-TPR profiles of all catalysts are presented in Figure 5. These profiles act as an important structural promoter during the reduction process, consistent with the BET surface area results shown in Table 4. Specifically, two distinct reduction peaks at 386 °C and 405 °C were observed for the Co/SiO_2_Al_2_O_3_ catalyst. These peaks were due to the reduction of Co_3_O_4_ to metallic Co [35]. The peak at 672 °C corresponded to the reduction of cobalt silicate. The peak shifted to a lower temperature after the addition of Al_2_O_3_ to the silica support [36]. This may be due to Al_2_O_3_ weakening the interaction between FeCo and SiO_2_. The H_2_-TPR profile of the FeCo/SiO_2_Al_2_O_3_ catalyst exhibited several overlapping reduction peaks between 350 °C and 425 °C, corresponding to the reduction of Co_3_O_4_ to metallic Co and Fe_3_O_4_ to FeO. A distinct peak at 648 °C was attributed to reduction of FeO to metallic Fe [46].

Table 4 summarizes the H_2_ consumption and reduction degrees (%) for all catalysts. The Co/SiO_2_Al_2_O_3_ catalyst showed both the highest H_2_ consumption and the highest reduction degree within the 150–400 °C range where Fischer–Tropsch synthesis (FTS) is typically carried out [47]. This indicates the presence of reducible metal oxide species and easier reduction of cobalt oxide nanoparticles, facilitating the formation of active cobalt sites for FTS.

### 3.5. Thermogravimetric Analysis and Differential Scanning Calorimetry (TGA–DSC) Analysis

**Figure 6:**
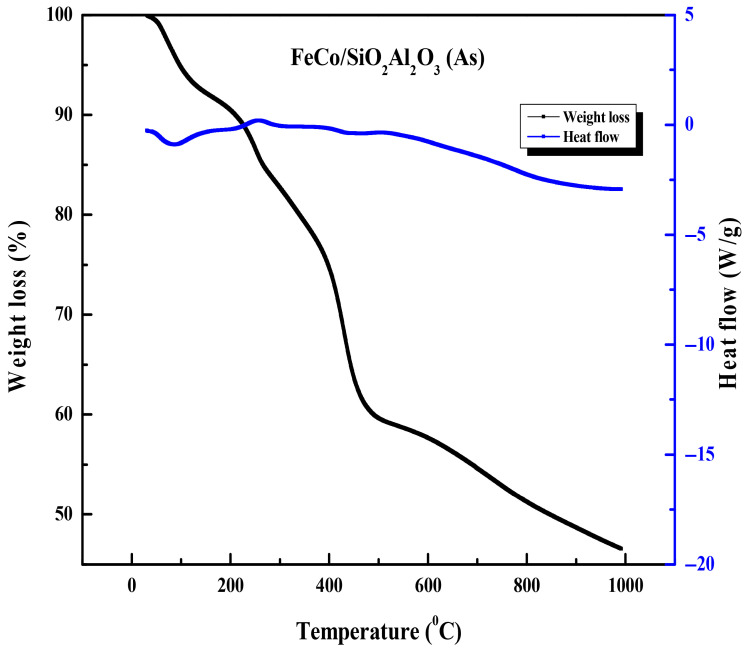
Thermogravimetric analysis and differential scanning calorimetry TGA-DSC thermograms of FeCo/SiO_2_Al_2_O_3_ (As) catalysts.

Figure 6 presents the TGA-DSC analysis of the FeCo/SiO_2_Al_2_O_3_ (As) catalyst. Here, “As” corresponds to as-synthesized catalyst (before calcination). The thermogram reveals five distinct weight loss stages, each corresponding to specific regions in the heat flow curve: from room temperature to approximately 200 °C, 200–300 °C, 300–400 °C, 400–500 °C, and 500–1000 °C. The initial weight loss up to 200 °C is primarily attributed to the removal of physically adsorbed water and residual solvents. There is consistent weight loss corresponding to a downward peak in the heat flow curve, representing an endothermic process, which is most likely the decomposition of a templating agent [48]. Between 200 °C and 300 °C, a consistent weight loss is observed, followed by a slight decrease in the rate of weight loss from 300 °C to 400 °C. This temperature range corresponds to an upward peak in the heat flow curve around 300 °C, indicating an exothermic event such as crystallization, which temporarily slows the weight loss. The weight loss continues between 300 °C and 400 °C, primarily due to the decomposition of the templating agent [48], reflected as an endothermic portion (negative slope) in the heat flow curve. Rapid weight loss occurs between 400 °C and 500 °C, coinciding with a local minimum in the heat flow curve, characteristic of an endothermic process such as melting or evaporation. This stage likely represents the major decomposition and removal of the templating agent. Finally, in the temperature range of 500 °C to 1000 °C, the weight loss curve continues to decline but at a reduced rate, while the heat flow curve remains linear with a negative slope. This slower weight loss suggests a diminished evaporation rate as most of the templating agent has already been removed by this stage.

**Table 2:** Crystal size calculation * based on the XRD data.

Catalyst	Avg. Fe_3_O_4_ Crystal Size (nm)	Avg. Co_3_O_4_ Crystal Size (nm)
**Co/SiO_2_Al_2_O_3_**	-	10.36
**FeCo/SiO_2_Al_2_O_3_**	16.19	16.35

* Using modified Scherrer equation [49].

**Table 3:** SEM-EDS analyses of synthesized catalysts.

Catalyst	Metal Loading (wt. %)				
	Fe	Co	Si	Al	O
Co/SiO_2_Al_2_O_3_	-	15.44	18.81	11.36	54.39
FeCo/SiO_2_Al_2_O_3_	18.47	22.07	9.16	4.95	45.35

**Table 4:** H_2_ consumption by different catalysts.

Catalyst	H_2_ Consumption (mmol/g)	Reduction Degree (%) *
Co/SiO_2_Al_2_O_3_	0.91	31.24
FeCo/SiO_2_Al_2_O_3_	0.73	14.39

* **Degree of reduction (%)** was defined, following the literature [45], as the ratio of the measured H_2_ consumption between 150 and 400 °C in the TPR peak to the theoretical H_2_ consumption (mmol H_2_/g) multiplied by 100.

### 3.6. Fourier Transform Infrared Spectroscopy (FTIR)

**Figure 7:**
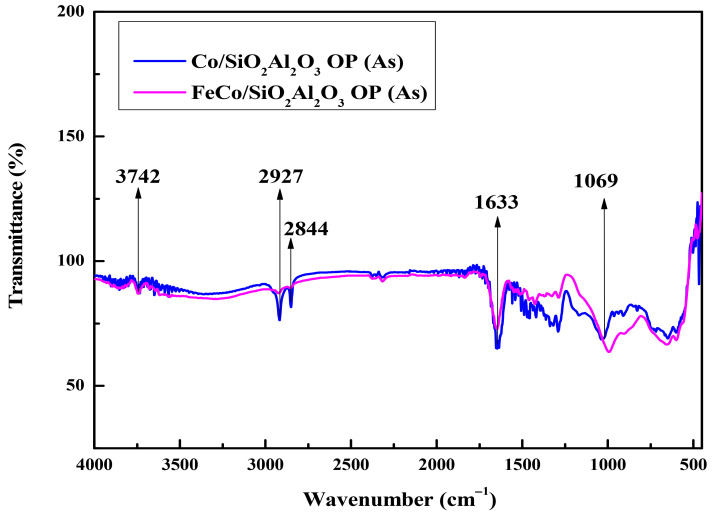
FTIR spectra of all catalysts prior to calcination: Co/SiO_2_Al_2_O_3_ (As) and FeCo/SiO_2_Al_2_O_3_ (As) catalysts.

The FTIR spectra of the as-synthesized samples are shown in Figure 7. The peak in the spectra at 565 cm^−1^ is due to the Fe-O stretching vibration [49].

FTIR spectroscopy confirmed the presence of a silica layer on the iron nanoparticles, as evidenced by a characteristic absorption band near 1069 cm^−1^ [50]. This band, originally located at 1100 cm^−1^, was observed to shift to lower wavenumbers, indicating a weakening of the siloxane (Si-O-Si) bridges. This shift is attributed to interactions between the silica and surface hydroxyl groups on the iron nanoparticles. However, these silica-related bands disappeared upon the addition of Al_2_O_3_ in the silica layer of the Co/SiO_2_Al_2_O_3_ (As) and FeCo/SiO_2_Al_2_O_3_ (As) catalysts. The band at 1633 cm^−1^ corresponded to the bending vibration of hydroxyl groups from interlayer water molecules [40,51]. Additionally, absorption bands at 2844 and 2927 cm^−1^ were attributed to symmetric and asymmetric C-H stretching vibrations from organic species [52]. The broad band between 3750 and 3000 cm^−1^, observed at 3742 cm^−1^, is assigned to O-H stretching from water adsorbed on silanol groups on the silica surface [53]. The IR spectra of all calcined catalysts are shown in the supplementary section of the manuscript (Figure S1 (See Supplementary file)).

### 3.7. X-Ray Photoelectron Spectroscopy (XPS)

**Figure 8:**
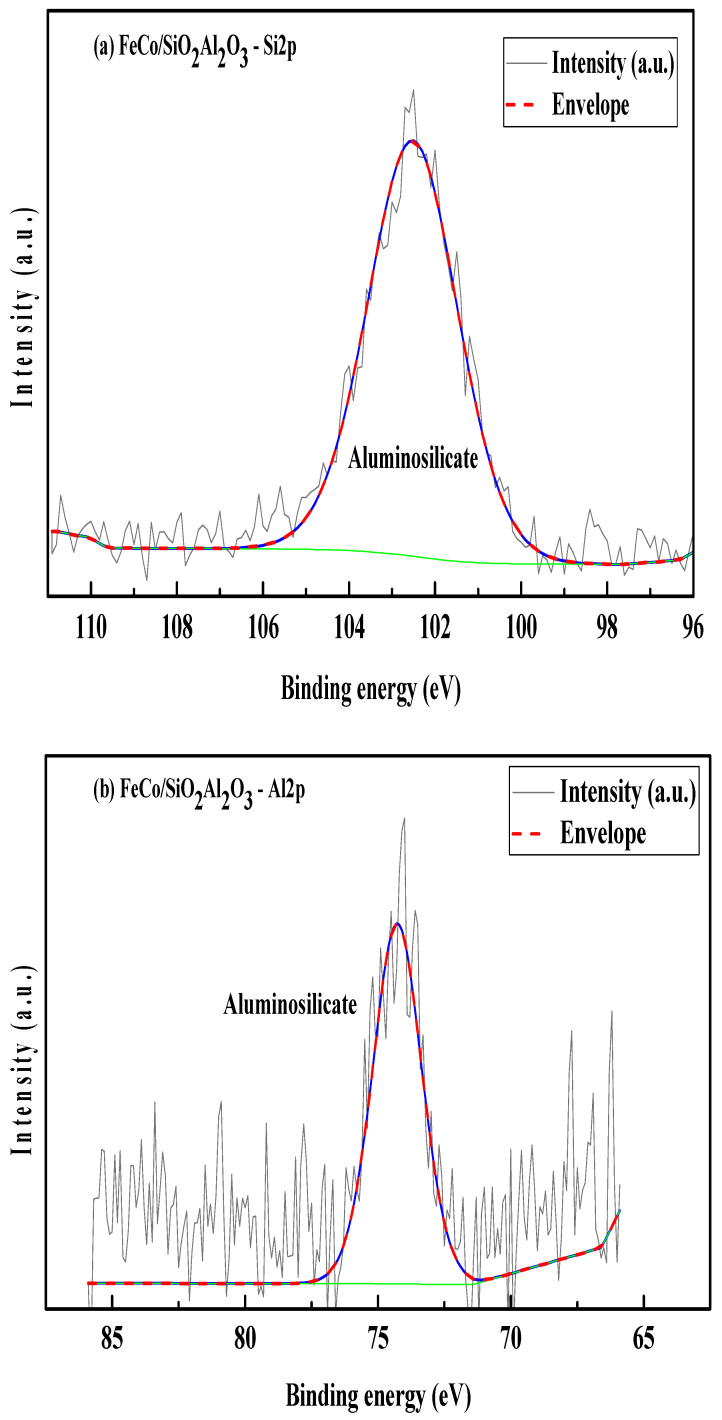

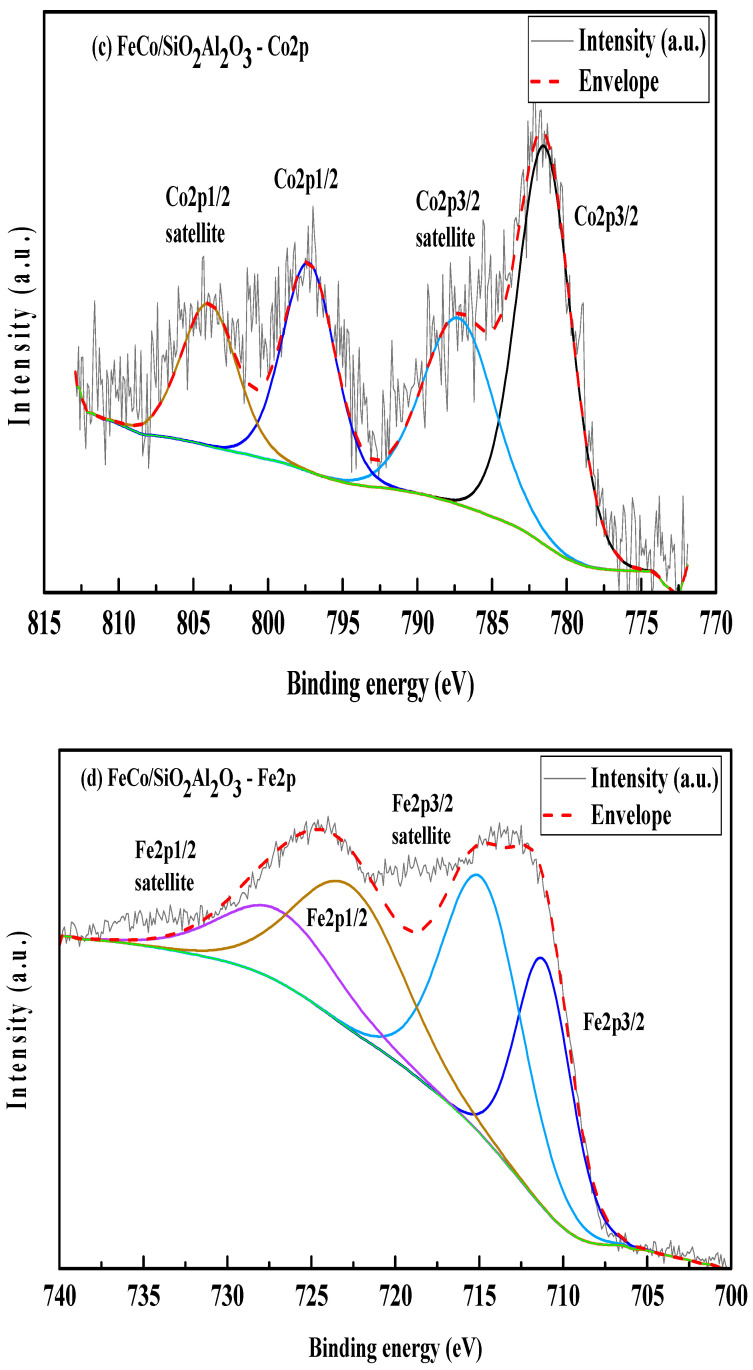
(**a**) Si2p, (**b**) Al2p, (**c**) Co2p, and (**d**) Fe2p XPS scans of FeCo/SiO_2_Al_2_O_3_.

X-ray photoelectron spectroscopy (XPS) was utilized to analyze the surface chemical composition and oxidation states of elements present in the FeCo/SiO_2_Al_2_O_3_ catalyst. Charge correction for all spectra was performed using the C 1s peak at 284.8 eV, corresponding to adventitious carbon (C–C bonds). The intensity in the spectra represents the measured value from the instrument, and envelope represents the resultant intensity from the deconvoluted peaks. Deconvolution of the C 1s spectrum revealed additional peaks attributed to oxygen-containing carbon species, including a component associated with O–C=O bonds, suggesting the presence of surface-bound carbon–oxygen functionalities [54]. Figure 8c shows the Si 2p XPS spectrum, featuring a single peak at a binding energy of 102.52 eV, confirming the presence of silicates in the FeCo/SiO_2_Al_2_O_3_ catalyst.

The Al 2p XPS spectrum of the FeCo/SiO_2_Al_2_O_3_ catalyst (Figure 8b) shows a binding energy peak at 74.27 eV, indicative of aluminum in an aluminosilicate framework, thereby confirming successful Al incorporation into the silica matrix. In the Co 2p region (Figure 8c), a positive binding energy shift of +3.29 eV is observed, attributed to the electronic influence of Fe, which possesses higher electronegativity. The Co 2p spectrum exhibits two principal peaks at 781.49 eV (Co 2p_3_/_2_) and 797.28 eV (Co 2p_1_/_2_), corresponding to spin–orbit splitting, along with satellite features at 787.23 eV and 803.95 eV, respectively. These features are characteristic of Co^2+^ species, confirming the presence of cobalt oxides in the catalyst matrix [54,55,56].

Two distinct peaks are observed at binding energies (BEs) of 781.61 eV and 797.25 eV, corresponding to Co 2p_3/2_ and Co 2p_1/2_, respectively (Figure 8c). Two satellite peaks are observed at 787.23 eV and 804.02 eV for the Co2p spectrum in the FeCo/SiO_2_Al_2_O_3_ catalyst.

The Fe 2p spectrum further supports the presence of iron oxides, showing a positive binding energy shift consistent with strong metal–metal interactions. Distinct multiplet-split peaks are observed at 711.16 eV (Fe 2p_3_/_2_) and 726.89 eV (Fe 2p_1_/_2_), accompanied by satellite peaks at 714.83 eV and 722.65 eV, respectively (Figure 8d). These spectral features indicate the coexistence of Fe^2+^ and Fe^3+^ oxidation states, confirming the formation of mixed-valent iron oxides within the catalyst [55].

### 3.8. Fischer–Tropsch Synthesis (FTS) of Fe-Based Catalysts

Fischer–Tropsch synthesis (FTS) reactions were conducted in a stainless steel microreactor over a temperature range of 200 °C to 350 °C to determine the optimum reaction temperature for CO conversion and hydrocarbon selectivity. The reactions were performed using a constant H_2_:CO molar ratio of 2:1, with a gas hourly space velocity (GHSV) of 12,000 h^−1^ and a pressure of 20 bar. The N_2_ gas flow rate was maintained at 1.5 mL/min. CO conversion and hydrocarbon selectivity were calculated using the equations below [57,58]. Although CO_2_ formation is important because of the water–gas shift reaction, it was not considered for the hydrocarbon selectivity calculation. The effect of reaction temperature on CO conversion and hydrocarbon selectivity for the Co/SiO_2_Al_2_O_3_ catalyst was discussed extensively in our previous paper [35].

$$X_{CO}\%=\frac{F_{CO,in}-F_{CO,out}}{F_{CO,in}}\times 100$$

$${CH}_{4}\;Selectivity\left(\%\right)=\frac{m{CH}_{4\;}}{m{CH}_{4\;}+2mC_{2}H_{6}+3m{C_{3}H}_{8\;}+4m{C_{4}H}_{10}}\times 100$$

$${C_{2}H}_{6}\;Selectivity\left(\%\right)=\frac{2m{C_{2}H}_{6\;}}{m{CH}_{4\;}+2mC_{2}H_{6}+3m{C_{3}H}_{8\;}+4m{C_{4}H}_{10}}\times 100$$

$${C_{3}H}_{8}\;Selectivity\left(\%\right)=\frac{3m{C_{3}H}_{8\;}}{m{CH}_{4\;}+2mC_{2}H_{6}+3m{C_{3}H}_{8\;}+4m{C_{4}H}_{10}}\times 100$$

In our previous study, we showed the effect of temperature on Co/SiO_2_Al_2_O_3_ catalyst activity for the FTS process [35].

**Figure 9:**
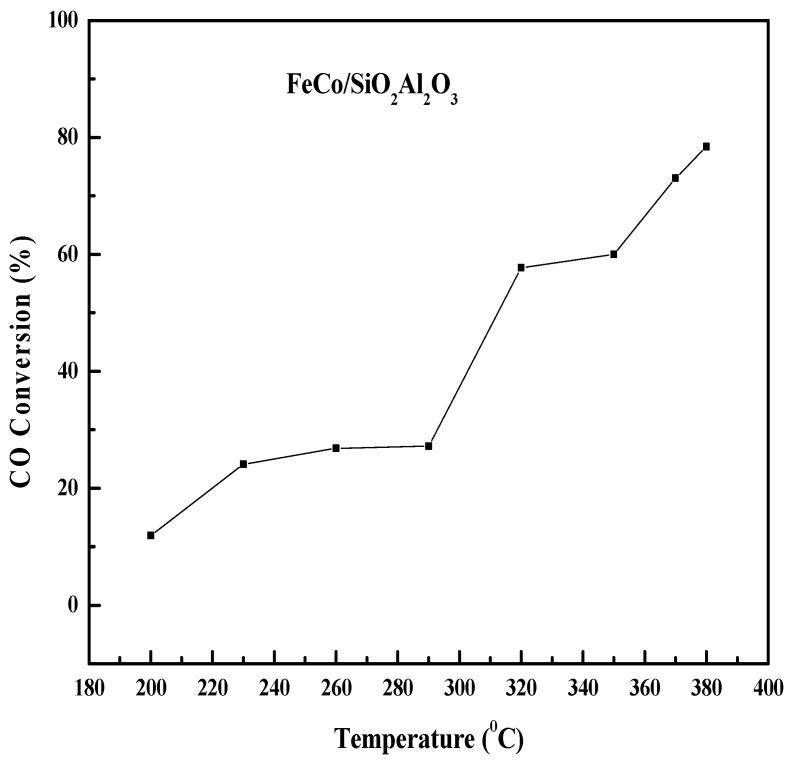

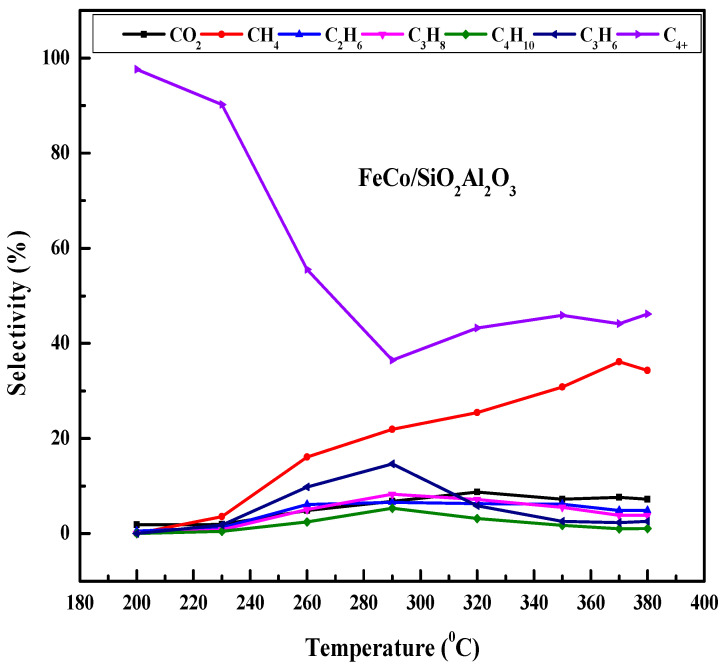
Comprehensive evaluation of the influence of reaction temperature on CO conversion and hydrocarbon product selectivity in Fischer–Tropsch synthesis using FeCo/SiO_2_Al_2_O_3_ catalyst. *Reaction conditions:* H_2_/CO = 2, pressure = 20 bar, GHSV = 12,000 h^−1^, N_2_ flow rate = 1.5 mL/min.

Figure 9 shows the effect of the reaction temperature on the CO conversion and product selectivity of the FeCo/SiO_2_Al_2_O_3_ catalyst. Initially, the CO conversion increased slowly up to 290 °C and then increased sharply to 57.7% at 320 °C. After that, the CO conversion increased further at higher temperatures. The CH_4_ selectivity increased up to 370 °C and then decreased [58]. The increasing trend in CH_4_ selectivity indicates that the catalyst was active for the methanation reaction. The presence of cobalt in the catalyst may facilitate the activation of CO_2_ (a byproduct of the FTS process), which can react with H_2_ to form CH_4_ [59]. At 290 °C, the selectivity for both CH_4_ (C_1_) and light hydrocarbons (C_2_-C_4_) reached their maximum, after which selectivity declined at higher temperatures (290–380 °C). In contrast, C_4_^+^ selectivity showed a decreasing trend with rising temperature up to 290 °C, then stabilized and remained nearly constant at higher temperatures. This is due to the higher reduction degree, lack of agglomeration of particles, and proper core–shell structure of this catalyst. The TPR, SEM, and TEM (Figure S2 (See Supplementary file)) results support the enhanced catalytic activity of this catalyst.

## 4. Time-on-Stream Studies of All Catalysts

To investigate the time-on-stream behavior of all catalysts, the Fischer–Tropsch synthesis (FTS) reaction was conducted for 30 h at 320 °C under identical operating conditions. In the case of the FeCo/SiO_2_Al_2_O_3_ catalyst, quite (60–65%) stable CO conversions of 65% and 61% were observed for 30 h.

**Figure 10:**
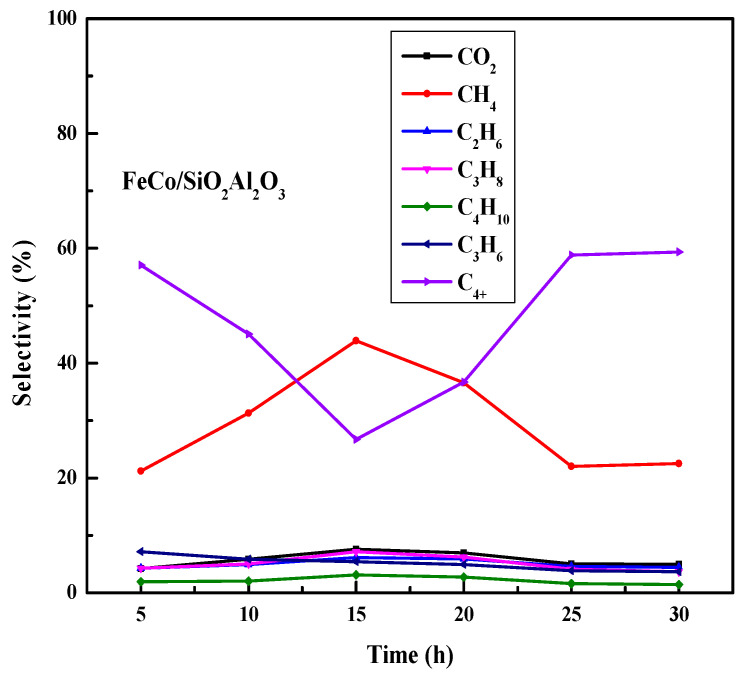
Time-on-stream performance of FeCo/SiO_2_Al_2_O_3_ catalyst during Fischer–Tropsch synthesis. *Reaction conditions:* H_2_/CO = 2, pressure = 20 bar, GHSV = 12,000 h^−1^, temperature = 320 °C, duration = 30 h, N_2_ flow rate = 1.5 mL/min.

As discussed in the H_2_-TPR experiments, the reduction of iron oxide to metallic iron generates more active sites, enhancing the FTS activity of the catalyst [60,61]. The catalyst structure effectively protects metallic Fe particles from oxidation and suppresses their growth during activation and reaction [62], making this catalyst favorable for the water–gas shift reaction. Methane selectivity increases during the first 15 h of reaction over the FeCo/SiO_2_Al_2_O_3_ catalyst, followed by a decrease thereafter (Figure 10). This trend suggests that the catalyst is initially active for methanation up to 15 h, after which CH_4_ formation reaches saturation and its selectivity declines. Correspondingly, C_4_^+^ selectivity rises after 15 h in our time-on-stream studies.

Overall, these results demonstrate that the core–shell catalyst structure promotes the formation of heavier hydrocarbons (C_4_^+^). Furthermore, the SiO_2_ and Al_2_O_3_ components in the catalyst support do not suppress surface basicity but instead provide active sites for mild hydrocracking reactions, including skeletal isomerization, hydrogenation, and double bond migration [60]. The acidic properties of SiO_2_Al_2_O_3_ supports, widely reported in the literature [61], likely contribute to the enhanced production of heavier hydrocarbons.

### 4.1. Spent Catalyst Characterization

#### 4.1.1. Scanning Electron Microscopy (SEM) Analyses

**Figure 11:**
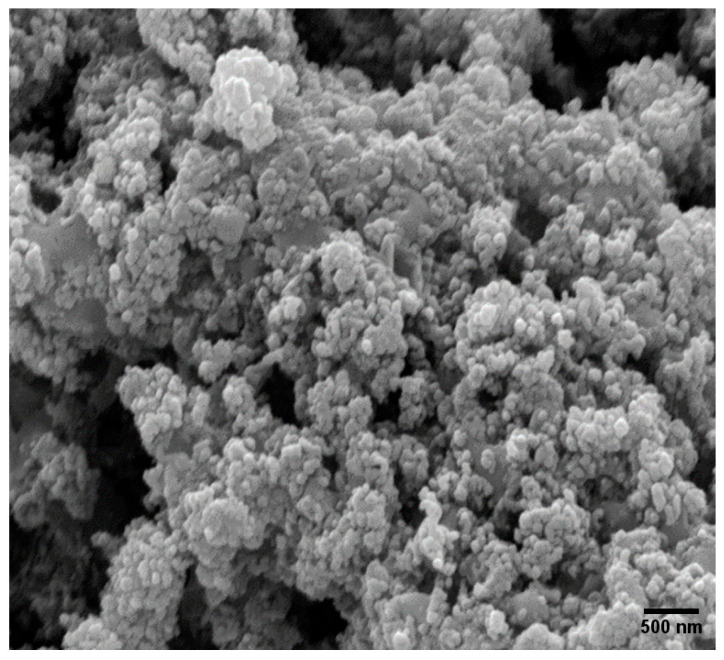
SEM images of spent FeCo/SiO_2_Al_2_O_3_ catalyst.

Figure 11 presents the SEM images of all spent catalysts, showing noticeable changes in surface morphology after the time-on-stream studies. Notably, particle agglomeration was observed in the FeCo/SiO_2_Al_2_O_3_ catalyst (Figure 11). Additionally, coke deposits were visible on the catalyst surfaces following the FTS reactions, which correlates with the observed decline in catalytic activity.

#### 4.1.2. Thermogravimetric and Differential Scanning Calorimetry (TGA-DSC) Analysis of Spent Catalysts

Thermogravimetric analysis coupled with differential scanning calorimetry (TGA-DSC) was performed on the spent catalysts to assess coke deposition and evaluate thermal stability. The measurements were conducted under an air atmosphere with a temperature ramp up to 1000 °C, allowing the detection of carbon oxidation events and thermal transitions associated with spent catalyst degradation [62,63,64].

The observed weight loss in the TGA profiles is primarily attributed to the combustion of carbonaceous species-such as coke and surface carbides—formed during the high-temperature Fischer–Tropsch reaction [48,62,63]. In some cases, a minor weight gain was also detected, which was attributed to the exothermic oxidation of residual reduced metal species upon exposure to air [49]. Given that all catalysts underwent hydrogen reduction prior to FTS, some degree of reoxidation during the TGA is expected.

Although the dominant trend was net weight loss due to coke combustion, it is important to acknowledge the potential presence of residual silicon carbide (SiC) in the spent catalyst samples. Despite implementing mechanical sifting steps to remove SiC particles, complete separation could not be guaranteed, and trace contamination may contribute to variability in the thermal analysis results.

**Figure 12:**
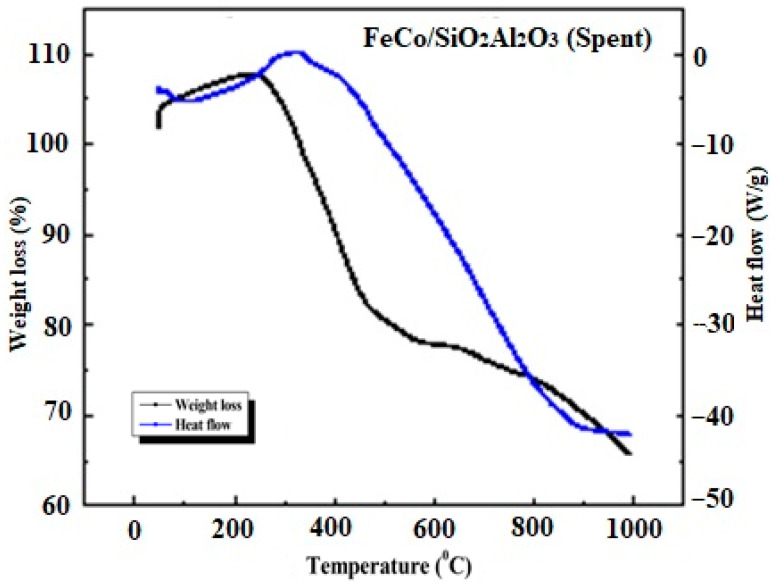
Thermogravimetric and differential scanning calorimetry (TGA-DSC) thermograms of spent FeCo/SiO_2_Al_2_O_3_ catalysts.

Figure 12 shows the TGA-DSC profiles of the spent FeCo/SiO_2_Al_2_O_3_ catalyst. As observed with other spent catalysts, a slight initial weight gain was observed, corresponding to an exothermic peak in the DSC curve, indicative of reoxidation of reduced metal species. This was followed by a substantial weight loss, comparable to that observed for the spent Fe_2_O_3_@SiO_2_ catalyst [35]. The major weight loss event aligns with an endothermic minimum in the DSC signal and is attributed to the oxidative combustion of carbonaceous deposits (e.g., carbides) accumulated during the Fischer–Tropsch reaction.

## 5. Conclusions

A 3D-printed stainless steel microchannel microreactor was employed to evaluate the catalytic performance of silica and alumina-supported core shell Fe and Co catalysts for Fischer–Tropsch synthesis (FTS). The XRD and SEM analyses confirmed good metal distribution in both Co/SiO_2_Al_2_O_3_ and FeCo/SiO_2_Al_2_O_3_ catalysts. The H_2_-TPR results further revealed that the synthesis method significantly affected the reducibility of metal oxides, thereby influencing the formation of active FTS sites that were consistent with the structural insights obtained from XRD and SEM studies. In addition, SEM-EDS analysis showed uniform metal ion distribution.

Catalytic activity tests were performed under FTS conditions in a 3D-printed microreactor at 20 bar [38]. While all catalysts showed a similar trend of increasing CO conversion with temperature, the FeCo/SiO_2_Al_2_O_3_ catalyst exhibited the highest performance, achieving a maximum CO conversion of 80%. Overall, the 3D-printed stainless steel microreactor demonstrated its effectiveness as a platform for catalyst screening and development, providing a scalable and practical solution to key challenges in Fischer–Tropsch synthesis.

## Figures and Tables

**Table 1:** BET analyses of Co/SiO_2_Al_2_O_3_ and FeCo/SiO_2_Al_2_O_3_ catalysts.

Catalyst	Surface Area (m^2^/g)	Pore Volume (cc/g)	Pore Diameter (nm)
**Co/SiO_2_Al_2_O_3_**	308	0.30	3.9
**FeCo/SiO_2_Al_2_O_3_**	241	0.41	6.5

## Data Availability

The data will be made available upon request.

## Supplementary Materials

The following supporting information can be downloaded at: https://www.mdpi.com/article/10.3390/molecules30173486/s1, Figure S1: FTIR spectra of Co/SiO_2_Al_2_O_3_ and FeCo/SiO_2_Al_2_O_3_ catalysts; Figure S2: Transmission Electron Microscopy (TEM) images of (a) Co/SiO_2_Al_2_O_3_ and (b) FeCo/SiO_2_Al_2_O_3_ core-shell catalysts.
