# Supplementary material for: Effect of Fe on Co-Based SiO2Al2O3 Mixed Support Catalyst for Fischer–Tropsch Synthesis in 3D-Printed SS Microchannel Microreactor
Source: Molecules. 2025 Aug 25;30(17):3486. doi: 10.3390/molecules30173486 (PMC12430590; doi:10.3390/molecules30173486)
Supplement: Supplementary file 1 [file molecules-30-03486-s001.zip › molecules-3761776-supplementary.pdf]

Effect of Fe on Co based $SiO_2Al_2O_3$ mixed support catalyst for Fischer-Tropsch Synthesis in 3D Printed SS Microchannel Microreactor

Meric Arslan[1], Sujoy Bepari[2], Juvairia Shajahan[3], Saif Hassan[2], Debasish Kuila[1, 2, 3]

1 Department of Applied Science and Technology, North Carolina Agricultural and Technical State University, Greensboro, North Carolina, 27411, United States

2 Department of Chemistry, North Carolina Agricultural and Technical State University, Greensboro, North Carolina, 27411, United States

3 Joint School of Nanoscience and Nanoengineering, North Carolina A&T State University, Greensboro, NC 27411, USA

*Corresponding author: dkuila@ncat.edu

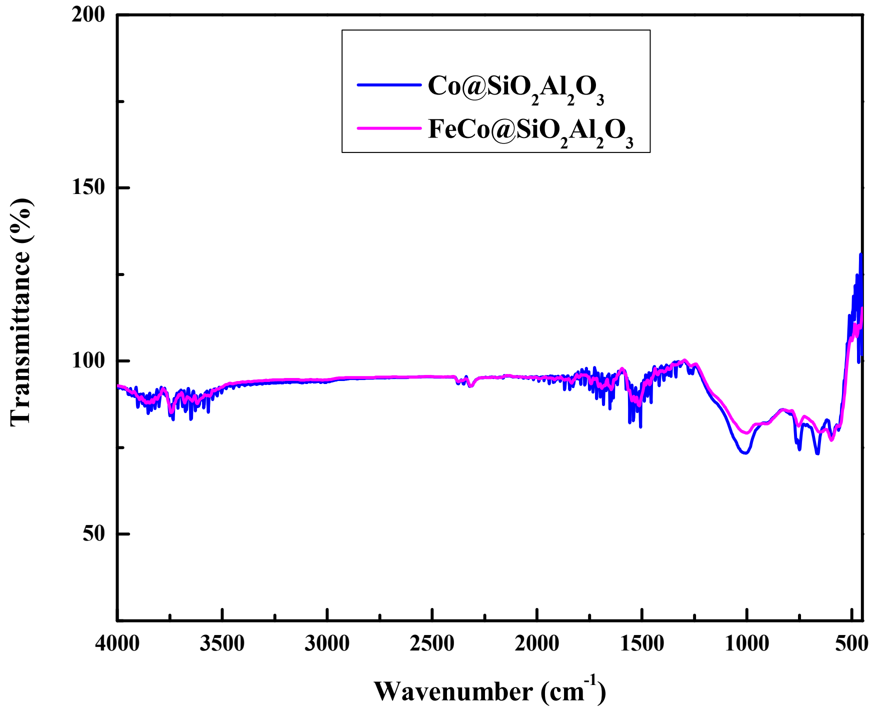

Fig. S1 FTIR spectra of Co/$SiO_2Al_2O_3$ and FeCo/$SiO_2Al_2O_3$ catalysts

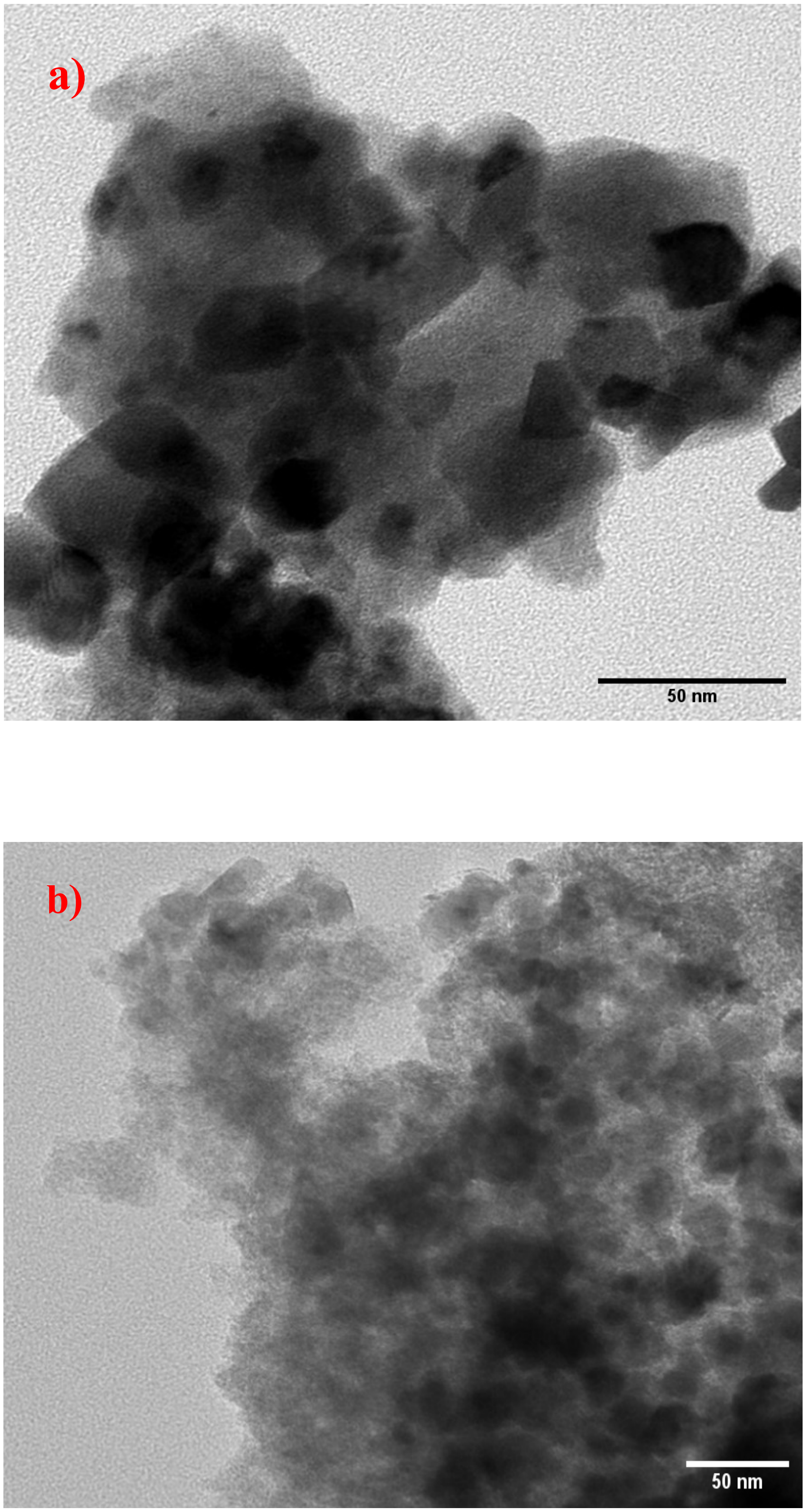

Fig. S2 Transmission Electron Microscopy (TEM) images of (a) Co/$SiO_2Al_2O_3$ and (b) FeCo/$SiO_2Al_2O_3$ core-shell catalysts
